# Recruiting and Conducting Online Dyadic Semi-Structured Interviews With LGBTQ+ Couples Facing Advanced Cancer

**DOI:** 10.1093/geroni/igab046.2238

**Published:** 2021-12-17

**Authors:** Kristin Cloyes, Lee Ellington, Brian Baucom, Katherine Supiano, Kathi Mooney, Sara Bybee

**Affiliations:** 1 University of Utah, Portland, Oregon, United States; 2 University of Utah, Salt Lake City, Utah, United States; 3 University of Utah Department of Psychology, Salt Lake City, Utah, United States; 4 University of Utah, Salt Lke City, Utah, United States

## Abstract

In this study, LGBTQ+ adult couples facing advanced cancer were recruited online. Eligible couples were sent a direct link to electronic consent and surveys in REDCap®. Participants were then invited to complete a 45-minute dyadic semi-structured interview regarding their experience of coping with cancer as a couple. This study faced difficulties in recruiting LGBTQ+ couples, and also faced the challenge of identifying and managing online responses from individuals misrepresenting themselves, and from automated accounts or “bots”. LGBTQ+ aging scholars must acknowledge how conducting research remotely with LGBTQ+ adults may necessitate changes in study design, such as changes to recruitment and more comprehensive eligibility screening designed to prevent and detect the collection of untrustworthy data. Ultimately, protecting the integrity of participant data in online research supports research accessibility and inclusion for LGBTQ+ older adults, and is the first step in conducting research that promotes health equity.

